# Complete Genome Sequence of Sponge-Associated Tenacibaculum mesophilum DSM 13764^T^

**DOI:** 10.1128/MRA.00517-19

**Published:** 2019-11-27

**Authors:** Sou Miyake, Melissa Soh, Yichen Ding, Henning Seedorf

**Affiliations:** a Temasek Life Sciences Laboratory, Singapore, Singapore; b Department of Biological Sciences, National University of Singapore, Singapore, Singapore; University of Maryland School of Medicine

## Abstract

Here, the complete genome sequence of sponge-associated Tenacibaculum mesophilum DSM 13764^T^ is presented. *T. mesophilum* is a close relative of the fish pathogen T. maritimum, which causes significant fish disease outbreaks in aquaculture facilities. The *T. mesophilum* genome sequence will serve as an important resource for comparative genomics approaches.

## ANNOUNCEMENT

*Tenacibaculum* species have often been found in association with marine hosts (1), such as fish and their eggs (e.g., Tenacibaculum soleae and Tenacibaculum ovolyticum) (1, 2), sponges (Tenacibaculum mesophilum) (1), and algae (Tenacibaculum amylolyticum) (1). It has also been reported that Tenacibaculum maritimum is the etiological agent of tenacibaculosis (3, 4), a common fish disease that causes great economic loss in aquaculture facilities around the world. *T. mesophilum* is a close relative of *T. maritimum*, and the genome sequence of *T. mesophilum* will be an important resource for comparative genomics with other *Tenacibaculum* species, in particular with *T. maritimum*. *T. mesophilum* DSM 13764^T^ (isolated from the sponge Halichondria okadai, collected in Numazu, Japan [1]) was obtained from DSMZ (Braunschweig, Germany) and grown in marine broth (BD Difco). Genomic DNA was prepared using two rounds of phenol-chloroform purification (modified according to Sambrook et al. [5]). A total of 10 μg of prepared genomic DNA was purified with AMPure XP magnetic beads, quality checked with a NanoDrop instrument and a Qubit fluorometer (Invitrogen, Carlsbad, CA, USA), and subsequently used for construction of PacBio RS II single-molecule and Illumina MiSeq paired-end (251 × 251-bp) libraries according to the manufacturer’s instructions. PacBio sequencing generated 79,714 reads with a total of 782,889,938 bases. Raw PacBio sequencing reads were corrected, trimmed, and *de novo* assembled into a single contig using default parameters in Canu 1.8 (6). MiSeq paired-end reads were used for further error correction using Pilon version 1.22 (7). As an initial quality check step of the assembly, RNAmmer version 1.2 (8) was used to annotate and verify the RNA genes. In addition, CheckM version 1.07 (9) was used to assess genome completeness and percent contamination and to find missing single-copy marker genes. The GenBank annotation of the genome was generated by the NCBI Prokaryotic Genome Annotation Pipeline (annotation software revision 4.6). The Pilon-polished assembly of *T. mesophilum* DSM 13764^T^ yielded a single contig for a total length of 3,358,580 base pairs, with a G+C content of 31.8% and an average coverage of 223× across the genome. A second assembly approach using the SMRT Analysis software (via HGAP4 [10]) with default settings, except that the estimated genome size was set as 3.44 Mb according to the *T. maritimum* genome available (11), resulted in a highly similar scaffold but remained unclosed, as one repeat region was not fully resolved by HGAP4. Pilon made corrections in 17 different locations of the Canu-assembled genome. The genome has an estimated completeness of 100% and contamination of 0.61% based on 457 marker genes conserved in *Flavobacteriaceae*, as identified by CheckM. No plasmid was identified. The Canu-assembled genome contained 3,053 genes, of which 75 were RNAs (15 rRNA, 55 tRNA, and 5 noncoding RNA [ncRNA] genes). The central metabolism of *T. mesophilum* DSM 13764^T^ is similar to that of *T. maritimum*, whose metabolic potential has been previously reported (11). A comparative analysis with other *Tenacibaculum* species will further help us to understand the ecological role of *Tenacibaculum* species in the marine environment and their contribution to fish disease.

### Data availability.

This whole-genome shotgun project has been deposited in GenBank under the accession numbers CP045192 (Canu assembly) and CP032544 (HGAP4 assembly). The version of the described Canu assembly in this paper is the first version, CP045192.1. PacBio RS II raw data are available under SRA accession number SRR9020974, and Illumina MiSeq raw data are available under SRA accession number SRR9020973.

## References

[1] Suzuki M, Nakagawa Y, Harayama S, Yamamoto S 2001 Phylogenetic analysis and taxonomic study of marine *Cytophaga*-like bacteria: proposal for *Tenacibaculum* gen. nov. with *Tenacibaculum maritimum* comb. nov. and *Tenacibaculum ovolyticum* comb. nov., and description of *Tenacibaculum mesophilum* sp. nov. and *Tenacibaculum amylolyticum* sp. nov. Int J Syst Evol Microbiol 51:1639–1652. doi:10.1099/00207713-51-5-1639. 11594591

[2] Pineiro-Vidal M, Riaza A, Santos Y 2008 *Tenacibaculum discolor* sp. nov. and *Tenacibaculum gallaicum* sp. nov., isolated from sole (*Solea senegalensis*) and turbot (*Psetta maxima*) culture systems. Int J Syst Evol Microbiol 58:21–25. doi:10.1099/ijs.0.65397-0. 18175676

[3] Avendaño-Herrera R, Toranzo AE, Magariños B 2006 Tenacibaculosis infection in marine fish caused by *Tenacibaculum maritimum*: a review. Dis Aquat Organ 71:255–266. doi:10.3354/dao071255. 17058606

[4] Masumura K, Wakabayashi H 1977 An outbreak of gliding bacterial disease in hatchery-born red seabream (*Pagrus major*) and gilthead (*Acanthopagrus schlegeli*) fry in Hiroshima. Fish Pathol 12:171–177. doi:10.3147/jsfp.12.171.

[5] Sambrook J, Fritsch EF, Maniatis T 1989 Molecular cloning: a laboratory manual. Cold Spring Harbor Laboratory Press, Cold Spring Harbor, NY.

[6] Koren S, Walenz BP, Berlin K, Miller JR, Bergman NH, Phillippy AM 2017 Canu: scalable and accurate long-read assembly via adaptive k-mer weighting and repeat separation. Genome Res 27:722–736. doi:10.1101/gr.215087.116. 28298431 PMC5411767

[7] Walker BJ, Abeel T, Shea T, Priest M, Abouelliel A, Sakthikumar S, Cuomo CA, Zeng Q, Wortman J, Young SK, Earl AM 2014 Pilon: an integrated tool for comprehensive microbial variant detection and genome assembly improvement. PLoS One 9:e112963. doi:10.1371/journal.pone.0112963. 25409509 PMC4237348

[8] Lagesen K, Hallin P, Rødland EA, Staerfeldt H-H, Rognes T, Ussery DW 2007 RNAmmer: consistent and rapid annotation of ribosomal RNA genes. Nucleic Acids Res 35:3100–3108. doi:10.1093/nar/gkm160. 17452365 PMC1888812

[9] Parks DH, Imelfort M, Skennerton CT, Hugenholtz P, Tyson GW 2015 CheckM: assessing the quality of microbial genomes recovered from isolates, single cells, and metagenomes. Genome Res 25:1043. doi:10.1101/gr.186072.114. 25977477 PMC4484387

[10] Chin C-S, Alexander DH, Marks P, Klammer AA, Drake J, Heiner C, Clum A, Copeland A, Huddleston J, Eichler EE, Turner SW, Korlach J 2013 Nonhybrid, finished microbial genome assemblies from long-read SMRT sequencing data. Nat Methods 10:563. doi:10.1038/nmeth.2474. 23644548

[11] Perez-Pascual D, Lunazzi A, Magdelenat G, Rouy Z, Roulet A, Lopez-Roques C, Larocque R, Barbeyron T, Gobet A, Michel G, Bernardet JF, Duchaud E 2017 The complete genome sequence of the fish pathogen *Tenacibaculum maritimum* provides insights into virulence mechanisms. Front Microbiol 8:1542. doi:10.3389/fmicb.2017.01542. 28861057 PMC5561996

